# An Efficient Segmentation and Classification System in Medical Images Using Intuitionist Possibilistic Fuzzy C-Mean Clustering and Fuzzy SVM Algorithm

**DOI:** 10.3390/s20143903

**Published:** 2020-07-13

**Authors:** Chiranji Lal Chowdhary, Mohit Mittal, Kumaresan P., P. A. Pattanaik, Zbigniew Marszalek

**Affiliations:** 1 Vellore Institute of Technology, Vellore 632014, India; chiranji.lal@vit.ac.in (C.L.C.); pkumaresan@vit.ac.in (K.P.); 2 Department of Information Science and Engineering, Kyoto Sangyo University, Kyoto 603-8555, Japan; 3 Telecom SudParis, 9 rue Charles Fourier, CEDEX 91011 Evry, France; ipriyadarshinipattanaik@gmail.com; 4 Faculty of Applied Mathematics, Silesian University of Technology, 44100 Gliwice, Poland

**Keywords:** virus, intuitionistic possibilistic fuzzy c-mean, support vector machine, segmentation, breast cancer, Mammography Image Analysis Society (MIAS) dataset, machine learning

## Abstract

The herpesvirus, polyomavirus, papillomavirus, and retrovirus families are associated with breast cancer. More effort is needed to assess the role of these viruses in the detection and diagnosis of breast cancer cases in women. The aim of this paper is to propose an efficient segmentation and classification system in the Mammography Image Analysis Society (MIAS) images of medical images. Segmentation became challenging for medical images because they are not illuminated in the correct way. The role of segmentation is essential in concern with detecting syndromes in human. This research work is on the segmentation of medical images based on intuitionistic possibilistic fuzzy c-mean (IPFCM) clustering. Intuitionist fuzzy c-mean (IFCM) and possibilistic fuzzy c-mean (PFCM) algorithms are hybridised to deal with problems of fuzzy c-mean. The introduced clustering methodology, in this article, retains the positive points of PFCM which helps to overcome the problem of the coincident clusters, thus the noise and less sensitivity to the outlier. The IPFCM improves the fundamentals of fuzzy c-mean by using intuitionist fuzzy sets. For the clustering of mammogram images for breast cancer detector of abnormal images, IPFCM technique has been applied. The proposed method has been compared with other available fuzzy clustering methods to prove the efficacy of the proposed approach. We compared support vector machine (SVM), decision tree (DT), rough set data analysis (RSDA) and Fuzzy-SVM classification algorithms for achieving an optimal classification result. The outcomes of the studies show that the proposed approach is highly effective with clustering and also with classification of breast cancer. The performance average segmentation accuracy for MIAS images with different noise level 5%, 7% and 9% of IPFCM is 91.25%, 87.50% and 85.30% accordingly. The average classification accuracy rates of the methods (Otsu, Fuzzy c-mean, IFCM, PFCM and IPFCM) for Fuzzy-SVM are 79.69%, 92.19%, 93.13%, 95.00%, and 98.85%, respectively.

## 1. Introduction

Over the estimated new cases of cancer in the USA for 2020, breast cancer is observed as the first leading cancer type in the female. In developing countries [1], there is a lack of early detection schemes, tolerable diagnosis, and cure facilities for breast cancer cases, so the survival rate is low compared with developed countries. Breast cancer needs to be detected at an early stage so that proper treatment can be given to reduce the rate of mortality. Small-sized calcium deposits are known as micro-calcification, and they are an indication of malignancy. Mammography is one of the best reliable diagnostics over other methods like Ultrasound, Positron emission tomography (PET), and Magnetic Resonance Imaging (MRI) [2]. Mammograms are unusual to determine the presence of benign or malignant disease with conviction. The radiologists recommend a patient to go for the next diagnosis in the cases of uncertainty. Opaque regions are typically noisy in digital mammogram images, and have poor contrast. For this reason, it is a challenging responsibility for the radiologist to detect and diagnose a cancerous region. The noise must also be eliminated before a mammogram is processed [3]. A significant amount of noise reduction algorithms have been developed in the last two decades. The Mammography Image Analysis Society (MIAS) Mini Mammographic Database is normally a database of mammograms used in such research work [4,5].

**Figure 1:**
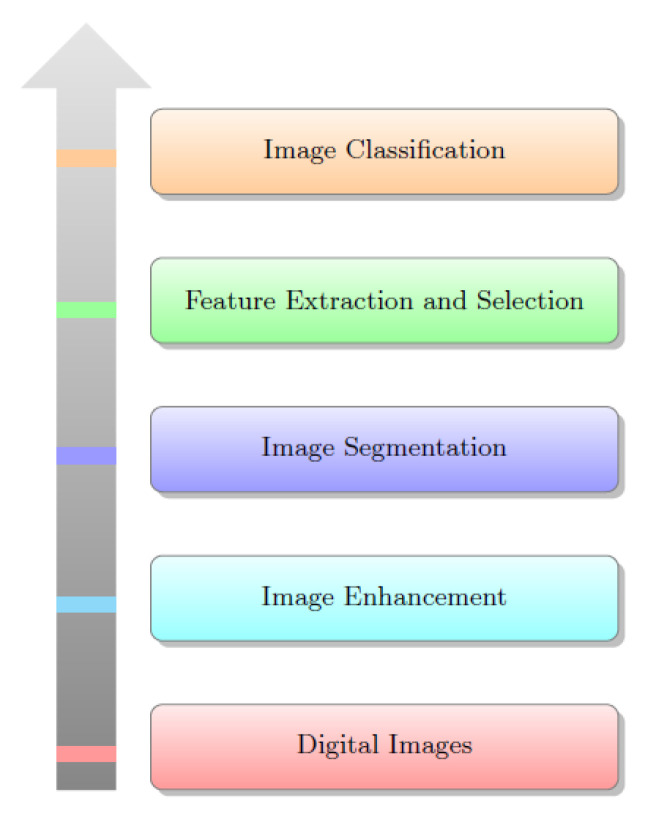
A simple block diagram for breast cancer detection steps.

The literature shows that the detection of viruses in breast cancer is highly inconsistent [6]. Figure 1 presents basic breast cancer detection procedures. The breast cancer detection method can be divided into five main approaches: (a) traditional image acquisition techniques; (b) Image enhancement model, especially for noise removal; (c) Find cancer affected area by detecting the suspicious region-of-interest (ROI) on medical images by using suitable segmentation method; (d) feature extraction; and (e) classification of benign or malignant from ROI. Proper segmentation is required for better feature extraction and classification. References [7,8,9] addresses many algorithms for the early detection of breast cancer detection. The evaluation of segmentation based on detection rate and accuracy gave the result of breast cancer detection cases [10,11,12]. The segmentation leads to feature extraction—the calculation of features based on density, texture, morphology, shape, and size of regions [13,14,15]. In the case of large and complex feature space with redundant and excessive features, there is a possibility to take excess time with a tendency to reduce accuracy in classification. It requires redundancy removal for performance improvement.

There are some cases where cancer is erroneously diagnosed among several patients. Osmanovic et al. [11] suggested a diagnostic method to resolve these cases by distinguishing between patients with and without breast cancer by defining the characteristics of cell nuclei present in an exceptional needle aspiration picture. Khwairakpam et al. [16] used fuzzy rules to identify the noise of images and filtered them using fuzzy weighted mean. They used genetic algorithm (GA) to optimize the parameters for fuzzy membership function. To evaluate the proposed filter edge-preserving factor and peak signal-to-noise ratio were used. Dutta et al. [17] proposed an approach to predict breast cancer by data mining methodologies, inference systems, and fuzzy logic. A novel prediction was proposed comprising of a fuzzy inference system with images collected from local clinics. This method performed better than other approaches like Logitboost [18], Locally Weighted Regression(LWL) [19], REP Tree [20], and so forth. Using deep learning algorithms, Khan et al. [21] used transfer learning methods for the classification and detection of breast cancer images. The feature extraction was executed with convolutional neural network (CNN) approaches like Residual Networks (ResNet), Visual Geometry Group Network (VGGNet), and GoogLeNet by feeding into a fully connected layer where malignant and benign cells are classified. Some researchers applied their work on classification and control problems by developing a fuzzy brain emotional learning neural network. Robio et al. [22] worked on self-organizing fuzzy modified least-square network. In References [23,24], the authors used discrete wavelet transform to analyze sub-bands within the Electroencephalography (EEG) parameter for creating model for epilepsy diagnosis. In References [25,26], the authors worked on parametric uncertainties and noisy outputs. Later they tested it for breast tumor classification and the chaotic system synchronization [27,28].

**Figure 2:**
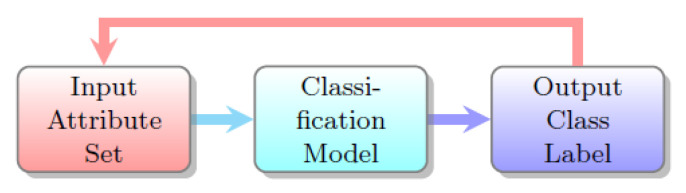
Classification Task.

Figure 2 shows a classification task related to the medical images as output. Classifiers methods like rough set data analysis, support vector machine (SVM), decision tree, neural network and linear discriminant analysis (LDA) were extensively used for medical image detection approaches [3,7,29,30].

The major contributions of the proposed method are the following:

1. In this paper, we considered the MIAS dataset for breast cancer detection.
2. For the detection of cancer in an image, we applied the existing segmentation techniques such as the Otsu algorithm, FCM (fuzzy c-mean) clustering, IFCM (ntuitionist fuzzy c-mean) clustering, and PFCM (possibilistic7fuzzy c-mean). After that, we propose a segmentation model, that is, IPFCM clustering. In addition to this, statistical Feature extraction techniques are also taken into account.
3. The simulation results are investigated with four classification models such as DT (decision tree), RSDA (rough set data analysis), FCM and Fuzzy SVM. Besides this, we considered IPFCM (intuitionistic possibilistic7fuzzy c-mean) clustering model and Fuzzy SVM classification model resulting in a more promising accuracy than state-of-the-art studies.
4. This paper presents the evaluation criteria of specificity, sensitivity, MCC (Matthew’s correlation coefficien), PPV ( positive predictive value), accuracy, and NPV (negative predictive value) for classification measurements.
5. Finally, simulation results of the proposed approach is fast and accurate recognition over existing results from the MIAS dataset.

The rest of paper is organized as follows. Section 2 discusses pre-processing (Section 2.1), segmentation (Section 2.2), clustering (Section 2.3) and feature extraction (Section 2.4). Section 3 highlights the overview of classifications methods and evaluation criterion. Section 4 describes the proposed Intutionistic possibilistic fuzzy clustering algorithm. Section 5 explains the setup used in the design of the experiment and the obtained results with respect to state-of-the-art methods. Finally, the paper is concluded in Section 6.

## 2. Materials and Methods

### 2.1. Basic Preprocessing: Noise Removal

Micro-calcification is the primary symptom of the malignant cells. In younger women who appear to indicate denser breast tissue, malignancy detection is particularly troublesome. Most mammography images are noisy and typically include regions of low contrast. Noise, like dust, hair, capture, and storage, are found in digital mammograms. Dense areas are typically noisy and have poor contrast in digital mammography images. For this reason, noise removal is required before breast cancer identification and treatment. Many noise removal algorithms have been proposed in the last two decades. Median filtering, max-min filter, midpoint filtering, adaptive median filtering, alpha-trimmed mean filtering, quantum noise filtering, impulse noise filtering, and wavelet thresholding are methods for mammogram noise removal.

### 2.2. Background of Segmentation

Segmentation is an essential technique for the analysis of image processing. Image Segmentation plays a crucial role in the diagnosis of disease. The segmentation of medical images aims to make digital images easier and more accessible to analyze. The image is partitioned in multiple non-overlapping, significant homogeneous areas during the segmentation process. Segmentation is based on the techniques of unsupervised clustering. It became challenging in the case of medical imaging due to poor contrast and the noise caused in the acquisition [31,32].

For better understanding and alleviation of the segmentation methods, many papers have been published in the last two decades. According to References [33,34,35], clustering has three main issues—(1) problem-solving, (2) decision-making, and (3) image segmentation. The threshold believed as the most straightforward method over other segmentation methods. The thresholding directive for segmentation is to use the Otsu algorithm [36] for medical images to maximize the class reparability for a class variance. The work in Reference [31] applied the fuzzy method in medical images to overcome the uncertainty issues of vagueness, boundaries, and variations in grey-level images. Clustering has the leading role in separating unlabelled data into discrete sets.

Several potential clustering methods are presented, including k-mean, fuzzy c-mean, artificial neural network, genetic algorithms, and many improved forms of such methods. However, it is challenging in designing an optimum solution from the thresholding technique for further improvement in disease detection methods by medical images; this was a call for constant effort from the research communities. The k-mean clustering method confines every data in a precise cluster which is not functional for all tenders. Meanwhile, the work in [31] employed Fuzzy c-mean for image segmentation which assigns each pixel to unlabeled fuzzy clusters so that each pixel was retained in all clusters with varying membership degrees. Motivated by this research effort, it is found that the membership of Fuzzy c-mean cannot reflect the degrees of data that belong to it. The potential of FCM clustering for diagnosing diseases like breast cancer has been proved to resolve the uncertainty and unknown noise in medical image segmentation.

Interestingly, fuzzy c-mean membership does not represent the degree of the belonging data. As one of the most effective solutions, the authors of References [37,38,39,40,41,42] used possibilistic c-means. Every element has value ranges of 0–1. For example, the possibilistic c-mean identifies outliers (noise points). Another solution in Reference [43] leveraged fuzzy-possibilistic c-mean algorithm to produce typicality values and membership values where clustering data are unlabeled. The authors of Reference [44] applied a possibilistic fuzzy c-mean algorithm that produces membership and possibility under useful point models or cluster centres. possibilistic fuzzy c-mean was useful in detecting fuzzy rule-structures. The work of Reference [45] implemented adaptive and non-adaptive fuzzy c-mean algorithms. The work in Reference [46] applied an adaptive approach to finding the weights of local spatial factors in local spatial continuity. The researchers applied MRIs and found substantial success with the proposed possibilistic fuzzy c-mean method. The possibilistic fuzzy c-mean method was more robust and efficient for many levels of noise. To overcome the noise condition drawback of fuzzy c-mean clustering, the authors of Reference [47] proposed an exponential fuzzy c-mean to enhance membership issues that results in a more meaningful membership degree over fuzzy c-mean.

The work of References [48,49,50] introduced the intuitionist fuzzy c-mean technique and used that approach in medical images. The key findings from this study of intuitionist fuzzy sets are—(1) the degrees of membership; (2) degrees of non-membership; and (3) degrees of hesitation. The goal of our proposed work is to hybridize two algorithms—(1) the possibilistic FCM algorithm, and (2) the intuitionist fuzzy c-mean algorithm. The traditional clustering methods cannot overcome various factors, like noisy data and outliers. Therefore, we used a possibilistic approach to solve those problems. In order to strengthen the possibilistic c-mean algorithm, we have hybridization with an intuitionist fuzzy c-mean algorithm. The Intuitionist fuzzy c-mean algorithm used to solve uncertainty issues by addressing degree of hesitation during the membership function [51,52,53].

### 2.3. Preliminaries of Clustering

The clustering methods were commonly used in the segmentation or classification of medical images. For many practical issues, clustering analyses were used to explore the data structure to understand the characteristics of data. Different clustering algorithms were proposed, including the Otsu algorithm [36], the k-means algorithm [54], the FCM algorithm [55], various improved FCM algorithms [43,44,45,46,47,48,49] and so on.

#### 2.3.1. Fuzzy C-Mean (FCM)

The FCM method [47] was demarcated a set *B* in *k* clusters. This batch has *N* members from $B=b_{1},b_{2},b_{3},...,b_{4}$. It was noticed that an uncertain state of the data $b_{1}$ was entrusted into several clusters by various degrees of membership $u_{lm}$. The membership of a cluster data is determined by paralleling its distance or dissimilarity from the cluster centroid $v_{m}$ to $d_{lm}$. Distance measurements are conducted with the aid of Euclidean formula defined in Equation (1):

(1) $$FCM=\sum_{m=1}^{k}\sum_{l}^{N}u_{lm}^{p}d_{lm}^{2},$$

where pε(1,∞) and $\sum_{m=1}^{k}d_{lm}=1$.

The task of the fuzzifier parameter *p* was to mechanize membership degree control over the objective function. The value of degree and centroid of membership was expressed in Equations (2) and (3), respectively.

(2) $$u_{lm}=\frac{1}{\sum_{q=1}^{k}\left(\frac{d_{lm}^{2}}{d_{lq}^{2}}\right)}$$

(3) $$v_{m}=\frac{\sum_{l=1}^{N}u_{lm}^{p}x_{l}}{\sum_{l=1}^{N}u_{lm}^{p}}$$

#### 2.3.2. Possibilistic Fuzzy C-Mean (PFCM)

Over k-mean results, the FCM clustering results are good but more noise sensitive. One limitation is that all membership degrees for each data point are incorporated cluster-wise into one, which leads to the abnormal points being members of clusters. The limitations of FCM were overcome by hybridization of the possibilistic approach with fuzzy c-mean, and that approach was named possibilistic fuzzy clustering (PFCM). Equation (4) represents the possibilistic fuzzy c-mean approach as:

(4) $$PFCM=\sum_{m=1}^{k}\sum_{l}^{N}u_{im}^{p}d_{lm}^{2}+\sum_{m=1}^{k}\lambda_{m}\left(\sum_{l}^{N}1-u_{lm}\right)$$

The role of membership degree and positive number were in Equations (5) and (6) as follows:

(5) $$u_{lm}=\frac{1}{1+\left(\frac{d_{lm}^{2}}{d_{lq}^{2}}\right)}$$

(6) $$\Lambda_{m}=W\frac{\sum_{l=1}^{N}u_{lm}^{p}d_{lm}^{2}}{\sum_{l=1}^{N}u_{lm}^{p}}$$

In Equation (6), *W* is an amendable weight which is typically set to one.

FCM (in Equation (3)) obtains the optimum solution for centroid revamping. In Equation (4), PFCM minimizes if all clusters are coincident clusters. The membership degree relies heavily on the gap between the data and the particular cluster without any consideration of other clusters.

#### 2.3.3. Intuitionistic Fuzzy C-Mean (IFCM)

Another enhanced fuzzy clustering was based on an intuitionistic fuzzy clustering algorithm. The traditional method of fuzzy c-mean was updated using intuitionist fuzzy sets. The cluster centers were modified so that intuitionist properties can be integrated with the fuzzy c-mean method. Atanassov suggested intuitionistic fuzzy sets [48] and discussed the presence of degree of hesitation. It cannot always be valid, according to the author, that the summation of membership degree and degree of non-membership is 1.There was a possibility for degree of hesitation. The level of hesitation was specified as 1 minus the total of degrees of membership and non-membership. The hesitation degree is as follows (Equation (7)):

(7) $$\pi_{A}=HesitationDegree=1-\left(MembershipDegree+non-MembershipDegree\right)$$

Initially, the hesitation degree was determined using Equation (8) and intuitionistic fuzzy membership values were obtained as follows:

(8) $$u_{lm}^{*}=u_{lm}+\pi_{lm},$$

where $u_{lm}^{*}$ denoted the intuitionistic fuzzy membership of the $m^{th}$ data in $l^{th}$ class. After replacing Equation (8) by Equation (9), the adapted cluster center will be:

(9) $$v_{m}^{*}=\frac{\sum_{l=1}^{N}u_{lm}^{*,p}x_{l}}{\sum_{l=1}^{N}u_{lm}^{*,p}}$$

In case of Equation (9), the cluster center was updated simultaneously with the membership matrix. The conventional k-mean clustering method has been used by many medical image segmentation systems proposed by different authors to classify tumors.

### 2.4. Feature Extraction

A medical image in the form of mammograms is segmented to extract the region of interest (ROI), followed by the feature extraction approach to identify significant features for deciding abnormal relentlessness to check whether tumor status is benign or malignant type. A common approach for the tumor detection of mammograms is using segmentation, followed by feature extraction and then classification to classify benign or malignant images [30]. The radiologists observe the results for identifying breast cancer from screening mammograms by extraction of categories of Breast Imaging Reporting and Data System (BI-RADS). The selection of significant features leads to proper classification. The vital features for extraction from mammograms are texture, shape, margin, and intensity. The segmentation for finding ROI isolates them in segmented area, foreground region of ROI area, and the background region of ROI.

Domineering formulas are encoded here for reckoning of features on the segmented areas. These feature are listed in Table 1.

Another feature of classifications of images is shown in Table 2. The disparity of the lesions, the intensity of the lesions, and their adjacent cells are dissimilar in the ROIs reaped from mammograms. Advanced lesions have considerably sophisticated gray values. In this exertion, the texture features of the mass and the back-ground regions are calculated through the gray level histogram measurements. These are mean, standard deviation, smoothness, skewness, uniformity, entropy, and kurtosis. The variables presumed for a convinced region $r_{rvin}$ are random variables to prompt the intensity, h(r) is the gray-level histogram, and *L* is the gray level.

## 3. Overview of Classifications Methods

Our proposed work is on noise removal in the preprocessing stage; Intuitionistic possibilistic Fuzzy c-mean clustering performed in the segmentation stage; significant features extraction by using statistical feature extraction methods; and final stage as Classification stage. Later classification results of SVM, decision tree, RSDA, and Fuzzy SVM are tested, optimized, and compared. These classification methods are as discussed below:

### 3.1. Decision Tree (DT)

A decision tree takes several correlations in factual lifespan, and it can be used in a range of machine learning applications [42,56], covering both classification and regression. The decision tree is aimed at visually and unambiguously representing decisions and decision making for decision analysis. A decision tree classification has three types of nodes—(1) root node, (2) splitting node, and (3) terminal node. Recently, the datasets are classified through the decision summary, well-defined via the tree in order. Then the respective class label is dispensed on the analysis with the terminal nodes, wherein the analysis cascades. A simple decision tree is shown in Table 3.

### 3.2. Rough Set Data Analysis (RSDA)

Rough set data analysis generates a set of rules from a system of decisions. A significant number of rules must be minimized. To get a minimum number of rules, it is essential to abstract the conditional attributes which are superfluous. Significant steps associated with rough set data analysis are core and reduct computation, finding the significance of attributes, constructing a decision table, producing rules, followed by classifying data [30]. To remove the more essential features, the core and the reduct are determined. Decision and core form the decision-making framework. A set of minimum rules can be created based on the decision system, and those rules are the basic building blocks of the classification model [27,38].

### 3.3. Support Vector Machine (SVM)

Vapnik proposed support vector machine concepts in Vapnik-Chervonenkis’ learning theory and structural risk minimization (SRM) inductive principle [8]. The SVM theory has attained abundant deliberation in earlier years.

Support Vector Machine provides a better performance in orthodox machine learning applications, pattern recognition for solving classification glitches. SVM is a valuable method for a nonlinear efficient approximation trick [39]. The support vector machine is primarily plotted to a high-dimensional feature space with the input data and leads to creating a spreadable hyperplane that exploits the margin in that space between two groups. The maximization of a margin between two groups can be assumed as a quadratic system designed to solve Lagrangian multipliers [40]. SVM uses the dot product functions to show the optimal hyperplane in the high-dimensional feature space known as kernels. For example, the optimal hyperplane elucidation is known as a combination of approximately input points, and they are called support vectors [41,57].

The inadequacy of the support vector machine is the sensitivity of the training procedure to the noises or outliers in the training datasets because of overfitting. Such uncertainty points are crucial to making decisions and creating an overfitting problem. The improved Fuzzy SVM is discussed in the subsequent sub-section.

### 3.4. Fuzzy SVM (FSVM)

Support vector machine classification has some drawbacks. Those drawbacks can be overcome by using fuzzy logic in SVM. FSVM [9] is a classification technique based on SVM exemplary for the classification of outliers or noise. The most challenging part of FSVM is acquiring the fuzzy membership of the training data. Lin and Wang projected a design process [9] for finding the fuzzy membership. The distance between the sample and its class center in the high-dimensional function space is used by a kernel extension development to measure a new fuzzy member.

Researchers suggested the ϵ-margin nonlinear classification prototype on the base of FCM clustering in the creative input space and the fuzzy If−Then rules. If−Then rule statements are used to formulate the conditional statements that comprise fuzzy logic. Another method proposed is a joint weight-based Fuzzy-SVM system [9], which reflects an identical training sample with various classes. The weight-based FSVM has difficulty in setting fuzzy membership values and diminishing computational complexity.

The basic theory is support vector machine [49] which is followed by a fuzzy support vector machine algorithm. Let *S* to be set of label m=l head training points for a binary classification delinquent is $\left(y_{m},z_{m},s_{m}\right)$ thru m=l inclines to. Their contribution data was obtainable by $y_{m}\in R^{n}$ accordingly specified a binary class label as $z_{m}\in \left\{-1,1\right\}$ and the fuzzy membership degree was $s_{m}\in \left[0,1\right]$ anywhere $y_{m}$ belongs to $z_{m}$. The binary classification delinquent model for a fuzzy support vector machine algorithm is essentially a discrimination restriction based quadratic programming problem which is given in Equations (10) and (11):

subject to

(10) $$z_{m}\left[\nu^{T}\delta \left(y_{m}\right)+t\right]\geq 1-\varpi_{i}$$

(11) $$\varpi_{i}\geq 0,m=1,\ldots ,l.$$

This quadratic-optimization problem is solved by building Lagrangian description and transforming it into the corresponding dual problem (Equations (12) and (13)):

(12) $$max_{\beta }\sum_{m=1}^{l}\beta_{m}\beta_{n}z_{m}z_{n}J\left(y_{m},y_{n}\right)s$$

subject to

(13) $$\sum_{m=1}^{l}\beta_{m}z_{m}=0$$

Consider $\beta_{m}$ as a Langrange multiplier through a value which is not equal to 0 when data point *m* is a support vector, and $J(y_{m},y_{n})$ is a kernel function.

During the use of Gaussian kernel function there is Equation (14)):

(14) $$J\left(y_{m},y_{n}\right)=e^{\left(-\frac{1}{2\sigma^{2}}||y_{m}-y_{n}|{|}^{2}\right)}$$

Now, the outcome of the solution by FSVM model for the class label of testing *y* can be expected as in Equation (15).

(15) $$z\left(y\right)=\left[\sum_{n=1}^{l}a\beta_{m}z_{m}J\left(y_{m},y_{n}\right)+t\right]$$

## 4. Proposed Intuitionistic Possibilistic Fuzzy Clustering

Medical images are complicated to understand. Therefore, it is required to remove undesirable portions of medical images. The noise removal process enhances the quality of the image.
**Algorithm 1:IPFCM Methodology.**InitializationCalculate PFCM which as follows:
(16)$$PFCM=\sum_{m=1}^{k}\sum_{l=1}^{k}u_{lm}^{P}d_{lm}^{P}+\sum_{m=1}^{k}\lambda_{m}\left(\sum_{l=1}^{N}\left(1-u_{lm}\right)\right)$$where, $$u_{lm}=\frac{1}{1+{\left(\frac{d_{lm}^{2}}{\lambda_{m}}\right)}^{\frac{1}{P-1}}}$$
$\lambda_{m}=W\frac{\sum_{l=1}^{N}u_{lm}^{P}d_{lm}^{2}}{\sum_{l=1}^{N}u_{lm}^{P}}$Hesitation degree is initially calculated with
(17)$\pi_{A}=HesitationDegree=1-\left(MembershipDegree+non-MembershipDegree\right).$Intuitionistic fuzzy membership value is attained by:
(18)$$u_{lm}^{*}=u_{lm}+\pi_{lm},$$where $u_{lm}^{*}$ signifies the intuitionistic fuzzy membership of the $m^{th}$ data in $l^{th}$ class.Substitute Equation (18) to Equation (16) for finding IPFCM.
(19)$IPFCM=\sum_{m=1}^{k}\sum_{l=1}^{k}u_{lm}^{*,p}d_{lm}^{P}+\sum_{m=1}^{k}\lambda_{m}\left(\sum_{l=1}^{N}\left(1-u_{lm}^{*}\right)\right)$ The improved cluster center will be: (20)$\lambda_{m}=W\frac{\sum_{l=1}^{N}u_{lm}^{*,P}d_{lm}^{2}}{\sum_{l=1}^{N}u_{lm}^{P}}$ and the cluster center was modernized and instantaneously the membership matrix was also rationalized.Accomplish the conclusion of iteration. Patronize the convergence standard.In case convergence was extended, break the iteration otherwise go back to Step 2.

**Figure 3:**
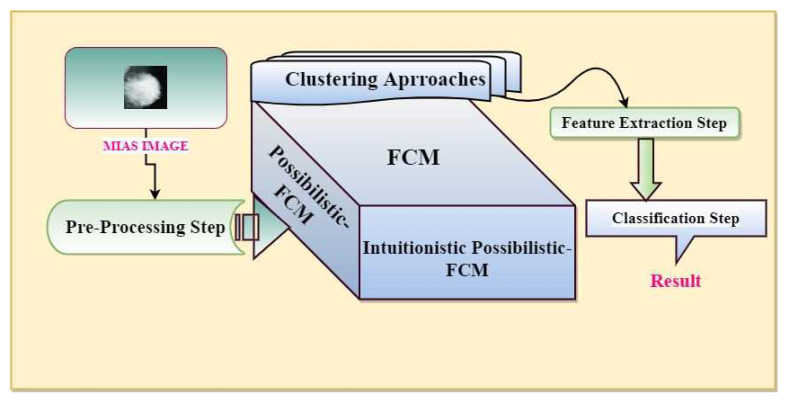
Schematic of the proposed method.

Possibilistic clustering approaches attempt to decrease the membership degree of noisy data, whereas the Intuitionist Possibilistic fuzzy clustering (IPFCM) approach assigns membership and non-membership degrees with hesitation degree. In Algorithm 1, the Intuitionist Possibilistic fuzzy c-mean methodology is written to strengthen the breast cancer detection system. Medical images are subsequently enhanced using Intuitionist Possibilistic fuzzy c-mean algorithms to form a cluster of pixels [47,48]. In order to improve membership assignments, a possibilistic approach has been used to overcome the noise cases. We also proposed an integrated intuitionistic fuzzy c-mean system [48,49] to improve the possibilistic c-mean algorithm [47]. A medical image segmentation system referred as an intuitionistic possibilistic fuzzy c-mean (IPFCM) clustering system. The design of the proposed system is in four stages as an initiative for pre-processing, main segmentation, that is, clustering, statistical extraction and final classification (Figure 3). The driving idea behind our proposed work is based on segmentation part that combines the possibilistic fuzzy c-mean with intuitionistic fuzzy c-mean and reduces the number of iterations to help with minimizing execution time.

## 5. Results and Discussion

The segmentation algorithm and classification methods are performed using MATLAB R2018a. At the classification stage, a support vector machine, decision tree, rough set data analysis, and fuzzy support vector machines are also executed to compare the accuracy of results.

### 5.1. Data Collection

The MIAS dataset [58] has a total of 320 digital mammogram images. These images are categorized into three types such as malignant, benign, and normal. There are 51 images in the malignant group, while 63 are benign. The remaining 206 images are normal. The pathological images are quite well known to be malignant. The original MIAS database was digitized at 50 micron-pixel edge, but reduced to 200-micron pixel edge and clipped in such a way that each image has (1024 × 1024) pixels.

### 5.2. Segmentation for Medical Imaging

**Figure 4:**
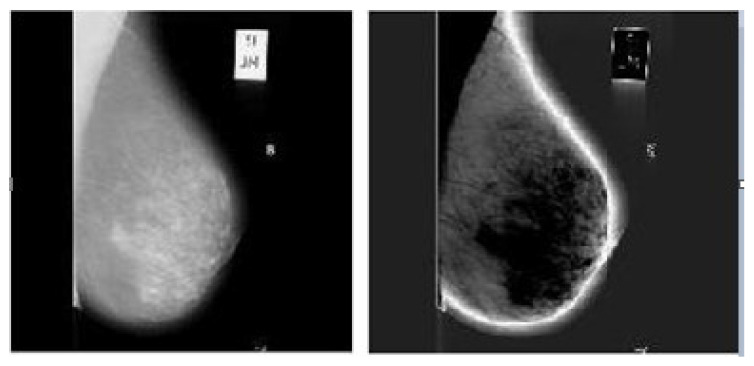
(**left**) Selected Input Image (**right**) Smoothened Image.

The selected input image is shown in Figure 4 left. Some noise removal algorithms are applied over input images are verified with few noise removal algorithms. We examined with median filtering, max-min filter, midpoint filter, adaptive filtering, adaptive-median filtering, alpha-trimmed-mean filter, quantum-noise filtering, impulse-noise filtering, and wavelet-thresholding methods for noise removal from mammogram input images. Figure 4 right presents a smoothened image per custom by Gaussian filter of diverse sizes (5×5) and standard deviation value 2.

A 5×5 window was preferred for calculating the average value of local gray-levels. The pixels are aimed at each point of the set. An average of the resemblance value to the reference images is kept on every 32 directions, and the points were devised the maximum spatial resemblance.

The interpolation made the segmentation of the MIAS image of the 32 contour points by using a polynomial interpolating method. The accuracy of the segmentation method is considered by superposing the contours perceived inevitably and manually to compute the transformation amid them.

### 5.3. Average Segmentation Accuracy

**Figure 5:**
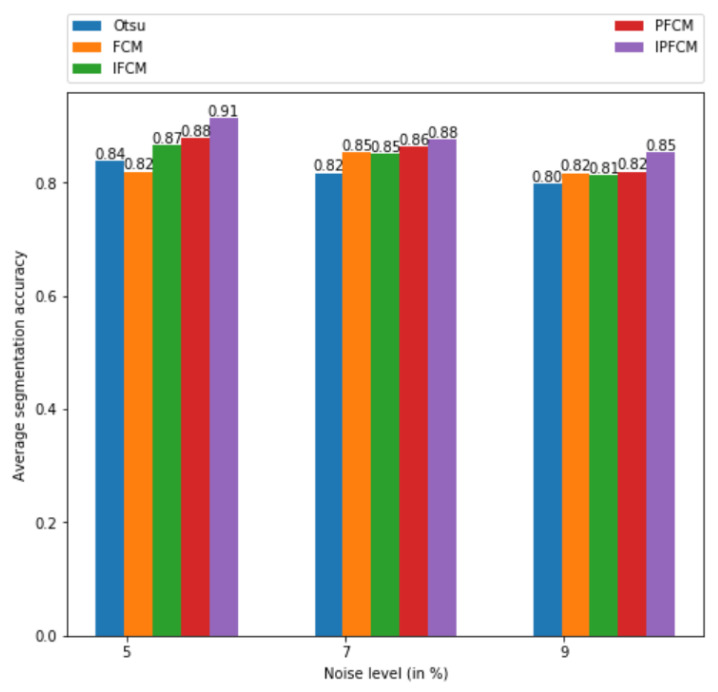
Average segmentation accuracy for Mammography Image Analysis Society (MIAS) images with different noise level chart.

With consideration of the optimal parameters for comparison of Otsu, FCM, IFCM, PFCM, and proposed IPFCM method for segmentation on the simulated MIAS breast cancer images as shown graphically in Figure 5. We also evaluated with different noise levels. Table 4 demonstrates the average accuracy of breast cancer segmentation for MIAS images with noise levels of 5%, 7%, and 9%. It was noted that the proposed approach with the negation function of Possibilistic is computationally inefficient compared to the negation function of Intuitionistic. The performance of the proposed IPFCM method with the hybridization of the negative function of Intuitionistic and the negative function of Possibilistic is better than the conventional segmentation methods.

**Figure 6:**
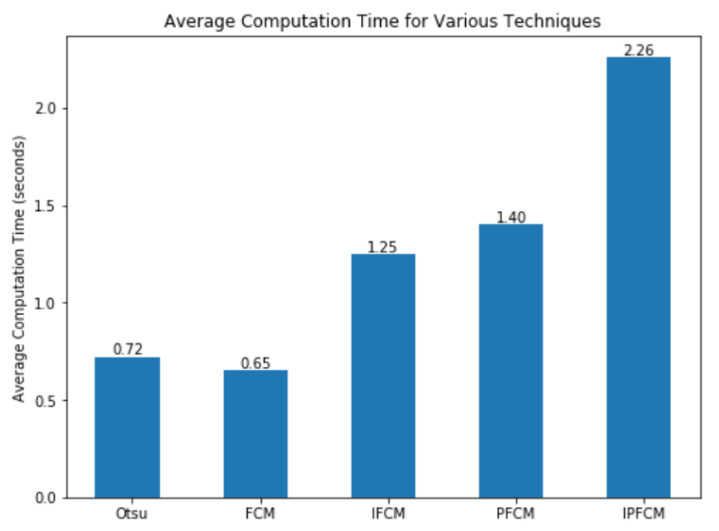
Average computation time for various techniques chart.

Table 5 and Figure 6 presents a comparison of the computation time it will take for various techniques for MIAS images.

### 5.4. Classification for Medical Imaging

We used the MIAS dataset for experimental classification. The efficiency of the SVM approach, decision tree, RSDA approach, and Fuzzy SVM approach is described in Table 6. The accuracy of the classification is more important for the diagnosis of breast cancer, then the consequences of an incorrect diagnosis that trigger unjustified surgery or even lead to death.

**Figure 7:**
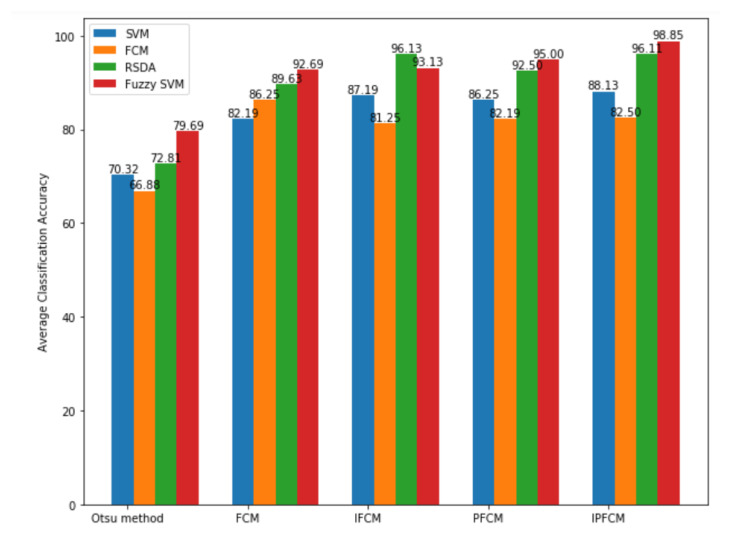
Comparative Classification average accuracy Chart.

The average classification accuracy rates of the methods (Otsu, FCM, IFCM, PFCM and IPFCM) for Fuzzy-SVM are 79.69%, 92.19%, 93.13%, 95.00%, and 98.45%, respectively (Table 6). It seems one-sided by using some features for Fuzzy-SVM to differentiate between benign and malignant breast tumors; it cannot accurately provide classification accuracy for each segmented image. For segmentation, the classification accuracy has changed accordingly and later going to the highest classification accuracy for IPFCM based FSVM classification. The best achieved classification accuracy rate is 98.45%. This proposed IPFCM segmentation with Fuzzy SVM method attains the uppermost classification accuracy rate (Figure 7).

### 5.5. Performance Evaluation

The specificity, sensitivity, Matthew’s correlation coefficient (MCC), positive predictive value (PPV), accuracy, receiver operating characteristic (ROC), and negative predictive value (NPV) were evaluated for classification measurements (Table 7).

The sensitivity and specificity are two statistical measures of the performance of a binary classification test. The confusion matrix supports research taking place in the root of actual and predicted results for positive actual and negative actual aftermaths. Consider the concern of positive actual outcome and fine two chances of predicted outcomes as “True Positive (*TP*)” and “False Negative (*FN*)”. Another consideration is of a negative actual outcome devising two predicted outcomes as “False Positive (*FP*)” and “True Negative (*TN*)”.

(21) $$\mathrm{Sensitivity}=\frac{TP}{TP+FN}$$

(22) $$\mathrm{Specificity}=\frac{TN}{TN+FN}$$

(23) $$\mathrm{Accuracy}=\frac{TP}{TP+FN}$$

(24) $$\mathrm{PPV}=\frac{TP}{TP+FP}$$

(25) $$\mathrm{NPV}=\frac{TN}{TN+FN}$$

(26) $$\mathrm{MCC}=\frac{TP\times TN-FP\times FN}{\sqrt{\left(TP+FP)(TP+FN)(TN+FP)(TN+FN\right)}}$$

Additionally, evaluation criterion “ROC curve” resolves the measurement for predictive accuracy for the suggested model. The “True Positive Rate (TPR)” and “False Positive Rate (FPR)” are designated in such a criterion. “AUC (Area under ROC Curve)” is castoff to compare the classifiers in two-class concerns.

**Figure 8:**
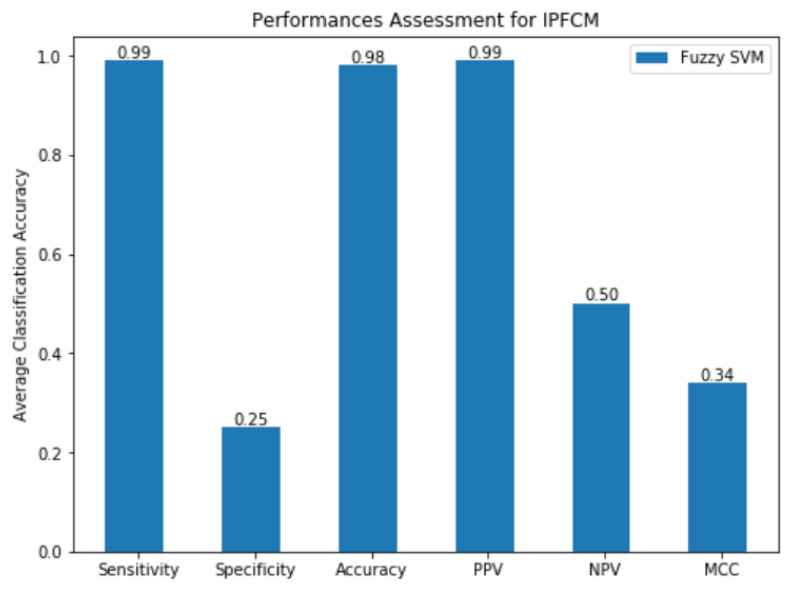
Performances Assessment for IPFCM.

The higher the values for sensitivity and specificity for the better performance of the system. In many cases, a higher sensitivity value can always be at a lower specificity value. SVM performed the worst, and the RSDA-based process performed better than SVM, which is only consistent with the parameter optimization performance. The decision tree-based method over matches the above techniques, but is still inferior to the Fuzzy SVM. The proposed approach achieves sensitivity-0.99, specificity-0.25, accuracy-0.98, PPV-0.99, NPV-0.50 and MCC-0.34, and has performed much better for Fuzzy SVM (with IPFCM segmentation) than the other classifiers (Table 8) and as shown graphically in Figure 8.

## 6. Conclusions and Future Research

Viruses can act as direct transforming agents and as triggering co-factors. Our research is conceived to detect digital mammograms. This machine conducts multiple-phase screening of breast cancer images. Noise and outliers trigger the low accuracy of the cluster analysis. In Fuzzy clustering, one data point was allocated to all clusters. As in Fuzzy’s clustering, the abnormal points were used by moving to other locations, which affected the centroids. Hence conventional fuzzy clustering like Fuzzy C-Means (FCM) is not sufficient to separate noise and outliers from typical results. But noise and outliers are not eliminated by the clustering method; hence they are forced to belong in one cluster due to general probabilistic constraint the amount of the membership degree of data across all clusters to 1. By incorporating the Possibilistic method, it enables the identification of outliers by the algorithm. In this paper, Intuitionist Possibilistic Fuzzy c-mean (IPFCM) not only minimizes the effect of outliers during the clustering process but also cesses it. These are also detected and extracted for further outlier mining. The detailed experiments show that IPFCM achieves reliable outlier detection results while maintaining the consistency of the clustering. The performance average segmentation accuracy for MIAS images with different noise levels 5%, 7%, and 9% of IPFCM is 91.25%, 87.50%, and 85.30% accordingly. The average classification accuracy rates of the methods (Otsu, FCM, IFCM, PFCM and IPFCM) for Fuzzy-SVM are 79.69%, 92.69%, 93.13%, 95.00%, and 98.85%, respectively. We conducted a comparison to compare our results with the most relevant studies, and the results are summarized in Table 9.

In future work, the segmentation and classification of this method with deep learning applications will be studied. In this way, breast cancer detection results can be obtained quickly and the application of this research is advanced.

## Figures and Tables

**Table 1:** Features for Segmentation.

Features	Explanation
Area $A_{r}$	Number of pixels in boundary area of ROI
Perimeter $P_{r}$	Number of pixels on boundary of ROI.
Circularity $C_{r}$	Number of pixels in boundary area of ROI. When there is a circular shape, circularity has a value of zero. By assuming $A_{r}$ for area and $P_{r}$ for the perimeter, circularity will be $C_{r}=1-\frac{4\pi A_{r}}{P_{r}^{2}}$.
Shape factor	Number of pixels on boundary of ROI. The count of burr around tumors will show the feature that is, region-of-interest as $S_{r}=\frac{P_{r}^{2}}{A_{r}}$. Assume $A_{r}$ for area, $P_{r}$ for the perimeter and $S_{r}$ for Shape factor.
Normalization radial length	$nrl\left(r\right)=\frac{rl(r)]}{max(rl(r))}$. Here, rl is radial length value meaning it is Euclidean Distance. So $rl\left(r\right)=\sqrt{{\left(b_{i}-t\right)}^{2}{\left(b_{j}-u\right)}^{2}}$. Here, $\left(b_{i},b_{j}\right)$ is centre position and (t,u) is boundary pixel position.
Mean-value of normalization based radial length	$nrl_{mean}=\frac{1}{P_{r}}\sum nrl(r)$
. Standard deviation value	$sigma=\sqrt{\frac{1}{P_{r}}\sum_{r=1}^{P}{\left(nrl\left(r\right)-nrl_{mean}\left(r\right)\right)}^{2}}$
. Entropy value	$E_{r}=\sum_{r=1}^{P_{r}}p_{k}\log p_{k}$. $p_{k}$ =probability of a certain nrl to the number of whole radials and $P_{r}$ perimeter.
The normalization value of central position shift	$NCPS=\frac{\sqrt{{\left(b_{i}-c\right)}^{2}+{\left(b_{j}-d\right)}^{2}}}{A}$. The pixels coordination position (c,d) is denoted with a minimum gray value inside the ROI. The Euclidian distance is calculated from the ROI centre $\left(c_{i}-c_{j}\right)$ at the position of the pixel with the lowest gray value, the ROI is divided.
Gradient	the gray value alteration among the boundary pixel and the 10^*th*^ pixel from this pixel with the radial direction $g_{r}=I\left(t,u\right)-I(i_{0},j_{0})$, Where I(t,u) is the gray value of the boundary pixel and $I(i_{0},j_{0})$ is the gray value of the 10^*th*^ radial pixel.

**Table 2:** Features for Classifications.

Features	Equations
mean of the intensity	$\sum_{rvin}^{L-1}r_{rvin}h\left(r_{rvin}\right)$
Standard deviation	$\sqrt{\sum_{rvin}^{L-1}{\left(r_{rvin}-mean\right)}^{2}h\left(r_{rvin}\right)}$
Smoothness	$1-\frac{1}{1+{\left(sigma\right)}^{2}}$
Skewness	$\sum_{rvin}^{L-1}{\left(r_{rvin}-mean\right)}^{3}h\left(r_{rvin}\right)$
Uniformity	$\sum_{rvin}^{L-1}h^{2}\left(r_{rvin}\right)$
Entropy	$\sum_{rvin}^{L-1}h\left(r_{rvin}\right)\log h\left(r_{rvin}\right)$
Kurtosis	$\sum_{rvin}^{L-1}{\left(r_{rvin}-mean\right)}^{4}h\left(r_{rvin}\right)$

**Table 3:** A Sample Decision Tree.

	Body pain	Cold	Vomiting	Fever
Image-1	High	Low	Yes	Yes
Image-2	Low	Low	No	Yes
Image-3	High	Low	Yes	No
Image-4	Low	Low	No	No

**Table 4:** Average segmentation accuracy with different noise level.

	Noise Level (in %)		
Segmentation Methods ↓	5	7	9
Otsu	0.8375	0.8156	0.7969
FCM	0.8187	0.8531	0.8163
Intuitionistic FCM	0.8656	0.85	0.8125
Possibilistic FCM	0.8781	0.8625	0.8188
**Proposed Intuitionistic Possibilistic FCM**	**0.9125**	**0.875**	**0.8531**

**Table 5:** Average computation time for various techniques in seconds.

Otsu	FCM	IFCM	PFCM	IPFCM
0.72	0.65	1.25	1.40	2.26

**Table 6:** Classification accuracy (Average) for five segmentation methods.

	Classification (All Features)			
Segmentation	SVM	Decision Tree	RSDA	Fuzzy SVM
Otsu	70.32	66.88	72.81	79.69
FCM	82.19	86.25	89.63	92.69
Intuitionistic FCM	87.19	81.25	96.13	93.13
Possibilistic FCM	86.25	82.19	92.5	95.00
**Proposed Intuitionistic Possibilistic FCM**	**88.13**	**82.5**	**96.1**	**98.85**

**Table 7:** Assessment Measures.

Evaluation Criterion	Definition
Sensitivity	The “Sensitivity” criteria is constructed on the positive circumstances of found results. The measurements are element of the perceived positive circumstances and the actual positive circumstances.
Specificity	The “Specificity” criteria is constructed on the negative circumstances of found results. The measurements are element of the perceived negative circumstances and the actual negative circumstances.
Accuracy	The “Accuracy” criteria is considered on the accuracy of found results. This criteria is the best common indicator which contributes the precision of forecast results.
PPV	“Positive Predictive Value” is approximately all the circumstances which calculate the decorously sensed positive circumstances concluded all sensed positive circumstances.
NPV	“Negative Predictive Value” is approximately totally the circumstances of conniving as the correctly noticed negative cases concluded totally detected negative circumstances.
MCC	One more operational accuracy evaluation display of machine learning methods is “Matthew’s Correlation Coefficient”. In the MCC, there is a comparison between the negative sample numbers and positive sample number led to finding unbalanced. The MCC compromises a virtuous evaluation ended the altogether accuracy.

**Table 8:** Performances Assessment for intuitionistic possibilistic fuzzy c-mean (IPFCM).

Evaluation Criterion	Classification (All Features)
	Fuzzy SVM
Sensitivity	0.99
Specificity	0.25
Accuracy	0.98
PPV	0.99
NPV	0.50
MCC	0.34

**Table 9:** Comparative results between the proposed work and the other related work.

Methodology ↓	Sensitivity	Accuracy
Crow Search Optimization based Intuitionistic Fuzzy Clustering [59]	0.98	0.96
Intuitionistic Fuzzy Rough Hybrid Technique [60]	0.97	0.98
Convolutional Network Method for Classifying Screening Mammograms [61]	0.97	0.95
Deep Neural Network with Support Value (DNNS) [62]	0.97	0.97
**Proposed (IPFCM and Fuzzy SVM)**	**0.99**	**0.98**
